# Demographics, Pattern of Care, and Outcome Analysis of Malignant Melanomas - Experience From a Tertiary Cancer Centre in India

**DOI:** 10.3389/fonc.2021.710585

**Published:** 2021-09-08

**Authors:** Jyoti Bajpai, George Abraham, Avanish P. Saklani, Anshul Agarwal, Sashanka Das, Ambarish Chatterjee, Akhil Kapoor, Prathyusha Eaga, Pradip Kumar Mondal, Arun Chandrasekharan, Prabhat Ghanshyam Bhargava, Sujay Srinivas, Siddharth Turkar, Bharat Rekhi, Nehal Khanna, Amit Kumar Janu, Munita Bal, Vikas Sureshchand Ostwal, Anant Ramaswamy, Jitender Rohila, Ashwin L. Desouza, Amrita Guha, Rajiv Kumar, Nandini Sharrel Menon, Sushmita Rath, Vijay Maruti Patil, Vanita Maria Noronha, Amit Prakashchandra Joshi, Siddhartha Laskar, Venkatesh Rangarajan, Kumar Prabhash, Sudeep Gupta, Shripad Banavali

**Affiliations:** ^1^Department of Medical Oncology, Tata Memorial Hospital, Homibhabha National Institute, Mumbai, India; ^2^Department of Surgical Oncology, Tata Memorial Hospital, Homibhabha National Institute, Mumbai, India; ^3^Department of Surgical Pathology, Tata Memorial Hospital, Homibhabha National Institute, Mumbai, India; ^4^Department of Radiation Oncology, Tata Memorial Hospital, Homibhabha National Institute, Mumbai, India; ^5^Department of Radiodiagnosis, Tata Memorial Hospital, Homibhabha National Institute, Mumbai, India; ^6^Department of Nuclear Medicine, Tata Memorial Hospital, Homibhabha National Institute, Mumbai, India

**Keywords:** malignant melanoma, LMICs, chemotherapy, immune checkpoint inhibitors, *BRAF*, Paclitaxel-carboplatin-LD interferon regimen, oral metronomic therapy

## Abstract

**Background:**

Treatment of malignant melanoma has undergone a paradigm shift with the advent of immune checkpoint inhibitors (ICI) and targeted therapies. However, access to ICI is limited in low-middle income countries (LMICs).

**Patients and Methods:**

Histologically confirmed malignant melanoma cases registered from 2013 to 2019 were analysed for pattern of care, safety, and efficacy of systemic therapies (ST).

**Results:**

There were 659 patients with a median age of 53 (range 44–63) years; 58.9% were males; 55.2% were mucosal melanomas. Most common primary sites were extremities (36.6%) and anorectum (31.4%). Nearly 10.8% of the metastatic cohort were BRAF mutated. Among 368 non-metastatic patients (172 prior treated, 185 de novo, and 11 unresectable), with a median follow-up of 26 months (0–83 months), median EFS and OS were 29.5 (95% CI: 22–40) and 33.3 (95% CI: 29.5–41.2) months, respectively. In the metastatic cohort, with a median follow up of 24 (0–85) months, the median EFS for BSC was 3.1 (95% CI 1.9–4.8) months versus 3.98 (95% CI 3.2–4.7) months with any ST (HR: 0.69, 95% CI: 0.52–0.92; P = 0.011). The median OS was 3.9 (95% CI 3.3–6.4) months for BSC alone versus 12.0 (95% CI 10.5–15.1) months in any ST (HR: 0.38, 95% CI: 0.28–0.50; P < 0.001). The disease control rate was 51.55%. Commonest grade 3–4 toxicity was anemia with chemotherapy (9.5%) and ICI (8.8%). In multivariate analysis, any ST received had a better prognostic impact in the metastatic cohort.

**Conclusions:**

Large real-world data reflects the treatment patterns adopted in LMIC for melanomas and poor access to expensive, standard of care therapies. Other systemic therapies provide meaningful clinical benefit and are worth exploring especially when the standard therapies are challenging to administer.

## Introduction

Melanoma is relatively rare in India, compared to other tropical countries. However, its incidence is rising globally (1). The incidence of melanoma depends on multiple factors such as age (more with advanced age), melanin content (inversely proportional) of the skin, latitude (more in tropical regions with increased UV exposure), altitude (more in higher altitude), and ethnicity (2).There are ethnic variations in the clinical and histopathological subtypes depending on the geography with superficial spreading and nodular subtypes common in Caucasians, while acral and mucosal predominantly are seen in Asians (3–6).

Melanoma cells are sensitive to T-cell mediated immune response mediated by tumor-infiltrated lymphocytes (TIL) due to the high tumor mutational burden caused by ultraviolet light exposure, cancer testis antigen expression, and mimicry of melanocyte lineage proteins (7), and these factors make melanoma a substrate for widespread utility of immune check point inhibitors and targeted therapy. The clinical feature of melanoma is a change in the size, color, and shape of the lesion making it distinct and different from the surrounding skin lesions (ugly duckling sign) (8). Although there is a marked increase in the diagnosis of localised melanoma due to overdiagnosis of stage 1 and 2 melanoma with the advances in screening and health care systems, there seems to be an increase in the incidence and diagnosis of metastatic melanoma also contributing to nearly 5% of the total melanoma cases (9). However, in the real-world scenario, there is under-documentation of the localised melanoma as treatment is sought at primary health care level and most patients present to tertiary care academic institutes with locally advanced or metastatic disease.

The 5 year survival for localised melanoma is nearly 99% but drops to 20% in the presence of upfront metastasis and highlights the importance of early diagnosis and treatment initiation (10). Historically, metastatic melanoma has a dismal prognosis with 5 year OS of approximately 10% (11). However, the advent of immune checkpoint inhibitors (ICI), including drugs that target programmed cell death 1 (PD-1) with or without inhibitors of cytotoxic T-lymphocyte–associated protein 4 (CTLA-4), *BRAF*, and MEK-targeted therapies, has improved the prognosis (1, 3–6, 12, 13) of metastatic melanoma with nearly half of patients on combination immunotherapy and one in three patients with targeted therapy surviving for 5 years (14).

The annual cost of melanoma treatment has increased exponentially by 288% in less than a decade, and it is expected to rise further with the advent of immune check point inhibitors and targeted therapies (2). The health care scenario in low-middle income countries (LMIC) has higher out-of-pocket expenditure (OOPE) from patients with low insurance coverage in the midst of low per capita income (15). Access to standard therapies is challenging in LMICs, and this has propelled research into other systemic therapies (ST) including chemotherapy, oral metronomic chemotherapy (OMCT), and low-dose subcutaneous interferons (LD-SC-IFN) with immunomodulatory properties. There is sparse data from real-world settings in India and merits exploration.

### Patients and Methods

Patients with histologically proven malignant melanomas, who presented to our tertiary care centre (blinded for peer review) between January 2013 and December 2019, were studied, including those with *de novo* or recurrent and/or metastatic disease.

Staging workup included MRI/contrast-enhanced (CE) CT scan of the affected primary site, whole-body F18 fluorodeoxyglucose positron emission tomography CECT (FDG PET-CECT), or X-ray and ultrasonography. Baseline demographic features, primary site, stage, histological details, mutation status, and treatment details were obtained from the electronic medical records. All patients were discussed in the multidisciplinary tumor board (MDT) after the staging and histopathology confirmation of melanoma. Localised melanoma was treated with surgery and adjuvant radiation for margin positivity or definitive radiotherapy with radical intent if deemed unresectable in the MDT. Locally advanced stage III cancers were given the option of adjuvant immunotherapy post-surgery. Locally advanced unresectable and metastatic melanoma patients were given the option of systemic therapy with immune check point inhibitors or targeted therapy (in *BRAF* positive patients). In patients who were not feasible for ICI or targeted therapies, palliative systemic therapy with chemotherapeutic agents were initiated based on the performance status and tolerance and clinical/radiological response was assessed every 2–3 month intervals.

### Ethics Statement

The study was conducted after approval from the Institutional Ethics Committee (IEC). Waiver of consent was obtained for retrospective study. All data were anonymized before the start of analysis.

### Statistical Analysis

Event-free survival (EFS) was defined as the time from the date of diagnosis until any event (including local or distant relapse/failures or progression or death). Overall survival (OS) was defined as the time from the date of diagnosis to death from any cause or last documented follow-up. Patients who were lost to follow-up were censored on the date of their last follow-up. Any radiological response was taken as response, and it was not strictly RECIST-based. Baseline host and tumor characteristics were correlated with survival outcomes. The data were analyzed using IBM SPSS Statistics for Windows, Version 24.0. Descriptive statistics were represented as median or percentage, and group comparisons were made using the χ2 test or Mann–Whitney U test, as appropriate. Survival was estimated using the Kaplan–Meier method and compared using the log-rank test.

## RESULTS

**Figure 1:**
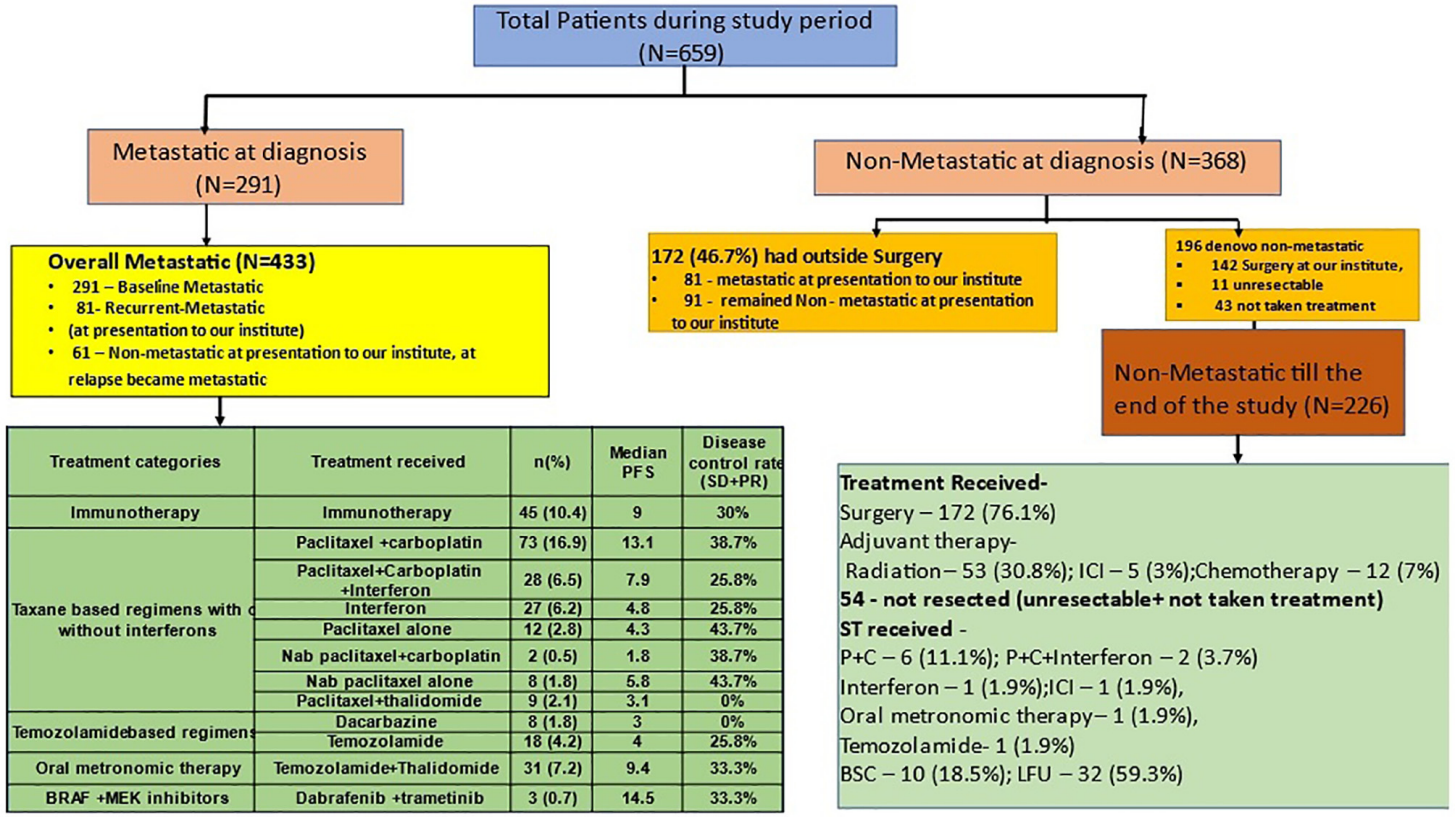
Patient cohorts for analysis and treatment patterns of patients with melanoma. **Figure 1** shows the distribution of patients with melanoma into the metastatic and nonmetastatic cohort which was used for subgroup analysis in the study. The green schematic boxes show the different modalities of treatment adopted in metastatic and non-metastatic setting with their median PFS. P+C, paclitaxel and carboplatin; Interferon, subcutaneous, low dose interferon; OMCT, oral metronomic chemotherapy; TMZ, temozolamide; BSC, best supportive care; PR, partial response; SD, stable disease; PD, progressive disease; CR, complete response; LFU, lost to follow up.

There were 659 patients; 368 (55.8%) were non-metastatic, while 291 (44.2%) were metastatic at the diagnosis. The median age was 53 (44–63) years; 388 (58.9%) were males and 271 (41.1%) were females. The commonest primary site was extremities 241 (36.6%) followed by anorectum 207 (31.4%); 364 (55.2%) were mucosal, while 295 (44.8%) were cutaneous melanomas. Forty-six patients were tested for *BRAF* mutation, out of which 5 (10.8%) were mutated (all positive *BRAF* cases were cutaneous melanomas), 1 (2.1%) was uninterpretable, and 40 (86.9%) were wild type (**Table 1** and **Figure 1**). The Eastern Cooperative Oncology Group (ECOG) performance status (PS) was 0 for 68 (10.3%), 1 for 252 (38.2%), 2 for 119 (18.1%), 3 for 78 (11.8%), and 4 for 39 (5.9%) of the patients. The performance status was undocumented in 103 patients (15.6%).

**Table 1:** Demographic characteristics of patients with malignant melanoma (N = 659).

Parameter	n	%
**Age in years (median, range)**	53 (IQR 44-63)	
<50	257	39.0
≥50	402	61.0
**Gender**		
Male	271	41.1
Female	388	58.9
**Site of Primary**		
Anorectal	207	31.4
Extremities	241	36.6
Head and neck	74	11.2
Ophthalmic	29	4.4
Genitals	38	5.8
Others	70	10.6
**Cutaneous *Vs*. Mucosal**		
Mucosal	364	55.2
Cutaneous	295	44.8
**Histopathology**		
Acral lentigous	31	16.7
Amelanotic	27	14.6
Epitheloid	12	6.5
Mixed	9	4.8
Nodular	77	41.6
Pigmented	4	2.16
Spindle	9	4.8
Superficial spreading	10	5.4
Others	6	3.2
Not specified	474	71.9

IQR, Interquartile range.

### Efficacy Outcomes

#### Overall Cohort

Among 659 patients with a median follow-up of 23.5 (0.5–86) months, 211 developed recurrences. Among these, 77 (36.5%) patients developed loco-regional recurrences, while 134 (63.5%) developed distant failures. After developing recurrences, 70 patients underwent surgery, 5 (7.1%) received adjuvant ICI and CT each, while 21 (27%) received RT. Second recurrence occurred in 17 (22%) patients (**Figure 1**).

**Figure 2:**
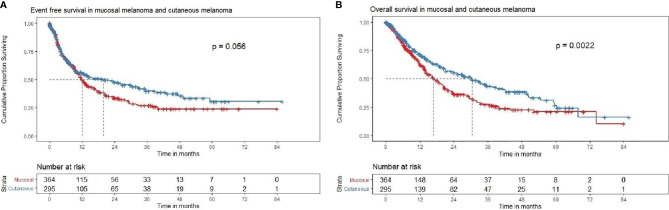
**(A)** Event-free survival in mucosal and cutaneous melanoma in the overall cohort (N = 659). Kaplan–Meier curve demonstrating the EFS in mucosal (red) and cutaneous (blue) melanoma in the overall cohort (N = 659). The median EFS of mucosal melanoma was 12.0 (95% CI 10.4–14.7), months and cutaneous melanoma was 19.8 (95% CI 12–33.3) months [HR = 0.81, (95% CI 0.65–1), P = 0.0567]. The 2-year EFS rate in mucosal and cutaneous melanoma was 33.9% (95% CI 28.4–40.6) and 47.4% (95% CI 41.1–54.8), respectively. **(B)** Overall survival in mucosal and cutaneous melanoma in the overall cohort (N = 659). Kaplan–Meier curve demonstrating the OS in mucosal and cutaneous melanoma in the overall cohort (N = 659). The median OS of mucosal melanoma was 16.9 (95% CI 14.1–20.3) months, and cutaneous melanoma was 30.3 (95% CI 23.2–41.2) months [HR = 0.69, (95% CI 0.55–0.87), P = 0.002]. The 2-year OS rate in mucosal and cutaneous melanoma was 36.9% (95% CI 31.1–43.9) and 55.3% (95% CI 48.7–62.8), respectively.

At a median follow-up of 23.5 months (0–86 months), the median EFS and OS are highlighted in **Table 2**. Patients with cutaneous melanomas fared better *versus* those with mucosal melanoma, with respect to EFS [HR: 0.80 (95% CI: 0.64–1.006, P = 0.057] and OS [HR: 0.69 (95% CI: 0.55–0.88, P = 0.01)] (**Table 3**, **Figures 2A, B**)

**Table 2:** Median EFS and OS according to primary site.

Site of melanoma	Median EFS (95 %CI) months	2 year EFS (95 %CI) percentage	Median OS (95 %CI) months	2 year OS (95 % CI) percentage
Mucosal melanoma	12 (10.4–14.7)	34% (28.4%- 40.6%)	16.9 (14.1–20.3)	37% (31.2%- 43.9%)
Cutaneous melanoma	20 (12–33)	47.5% (41.1%- 54.8%)	30 (23.2–41.2)	55.3% (48.7%- 63%)
Extremity melanoma	23.6 (12.5 – 35.4)	49.1% (42.1% – 57.4%)	32.5 (25.2 – 42.14)	57.9% (50.6 – 66.3%)
Anorectum	10.4 (9.1 – 12.3)	25.5% (18.8 %– 34.4%)	14.1 (12.3 – 17)	27.4% (20.4% - 36.8%)
Other sites including head and neck/soft tissue	14 (12 – 26)	43.5% (36.2 %– 49.1%)	21.8 (18.6 – 31.9)	47.6% (40%- 56.6%)

**Table 3:** Univariate and multivariate analysis for EFS and OS (only significant factors).

Univariate and Multivariate analysis for EFS (Only significant factors)												
	Overall cohort (N = 659)			Baseline Non-Metastatic (N = 368)			Baseline Metastatic (N = 291)			Overall Metastatic (baseline + relapsed) (N = 433)		
	N	Univariate	Multivariate	N	Univariate	Multivariate	N	Univariate	Multivariate	N	Univariate	Multivariate
**Prognostic factor**	** **	**HR (95%CI); P value**			**HR (95%CI); P value**		** **	**HR (95%CI); P value**		** **	**HR (95%CI); P value**	** **
**Site of Primary**												
Anorectal	207	(Ref.)	(Ref.)	91	(Ref.)		116			160		
Extremities	241	0.68 (0.52-0.88) ;0.003	0.83 (0.63-1.08) ;0.165	156	0.82 (0.56-1.22) ;0.334		85	0.74 (0.52-1.07) ;0.112		147	0.82 (0.62-1.10) ;0.181	
Others	211	0.71 (0.55-0.93) ;0.012	0.74 (0.57-0.97) ;0.029	121	0.81 (0.54-1.22) ;0.312		90	0.74 (0.52-1.06) ;0.102		126	0.89 (0.67-1.20) ;0.452	
**Surgery**												
No	308	(Ref.)	(Ref.)	64	(Ref.)							
Yes	351	0.34 (0.27-0.43) ;0.000	0.34 (0.27-0.43) ;0.000	304	0.42 (0.28-0.62) ;0.000							
**Mucosal *Vs*. Cutaneous**												
Mucosal	364	(Ref.)	(Ref.)	185	(Ref.)		179			248	(Ref.)	(Ref.)
Cutaneous	295	0.81 (0.65-1.01) ;0.057		183	0.84 (0.61-1.16) ;0.284		112	0.91 (0.67-1.24) ;0.555		185	0.89 (0.69-1.13) ;0.336	
**Therapy offered**												
BSC	123			50			73			110		
Any ST	305	0.77 (0.58-1.00) ;0.052		123	0.99 (0.63-1.55) ;0.959		182	0.58 (0.41-0.81) ;0.001		264	0.89 (0.67-1.18) ;0.404	
**Univariate and Multivariate analysis for OS (Only significant factors)**												
**Site of primary**												
Anorectal (Ref.)	207			91			116			160		
Extremities	241	0.55 (0.42-0.72) ;0.000	0.65 (0.36-1.20) ;0.168	156	0.71 (0.47-1.08) ;0.109		85	0.62 (0.42-0.92) ;0.016	0.71 (0.47-1.08) ;0.110	147	0.60 (0.45-0.82) ;0.001	0.49 (0.26-0.91) ;0.024
Others	211	0.63 (0.48-0.84) ;0.001	0.73 (0.51-1.05) ;0.090	121	0.80 (0.52-1.24) ;0.320		90	0.62 (0.43-0.90) ;0.011	0.66 (0.45-0.99) ;0.045	126	0.75 (0.55-1.02) ;0.067	0.73 (0.50-1.07) ;0.104
**Therapy offered**												
BSC	123	(Ref.)	(Ref.)	50			73			110	(Ref.)	(Ref.)
Any ST	305	0.48 (0.37-0.64) ;0.000	0.46 (0.35-0.61) ;0.000	123	0.60 (0.38-0.95) ;0.031	0.55 (0.35-0.88) ;0.013	182	0.32 (0.22-0.46) ;0.000	0.35 (0.24-0.50) ;0.000	264	0.41 (0.32-0.53) ;0.000	0.54 (0.40-0.72) ;0.000
**Surgery**												
No	308	(Ref.)	(Ref.)	64	(Ref.)							
Yes	351	0.28 (0.22-0.35) ;0.000	0.40 (0.30-0.53) ;0.000	304	0.34 (0.22-0.51) ;0.000	0.38 (0.20-0.73) ;0.004						
**Mucosal *Vs*. Cutaneous**												
Mucosal	364		(Ref.)	185			179			248		(Ref.)
Cutaneous	295	0.70 (0.55-0.88) ;0.002	1.05 (0.63-1.75) ;0.849	183	0.74 (0.53-1.04) ;0.082		112	0.82 (0.59-1.13) ;0.220		185	0.70 (0.54-0.90) ;0.006	1.12 (0.66-1.91) ;0.669

Ref, reference category; BSC, best supportive care; ST, Systemic therapy.

The median and 2-year EFS for mucosal group were found to be 11.9 (95% CI 10.3–14.3) months and 32.2% (95% CI 26.5–39.2%) respectively. For cutaneous group, median and 2-year EFS were 19.8 (95% CI 11.6–33.3) months and 47.7% (95% CI 41.4–55.1%), respectively (**Figure 2A**). For Uveal group, median and 2-year EFS were 13.8 (95% CI 6.8–NA) months and 49.7% (95% CI 33.3–74.9%), respectively.

The median OS and 2-year OS for mucosal group were 16.8 (95% CI 14.1–19.9) months and 34.4% (95% CI 28.4–41.6%), respectively. For cutaneous group, median and 2-year OS were 30.3 (95% CI 23.2–41.2) months and 55.6% (95% CI 49.0–63.2%), respectively (**Figure 2B**). For uveal group, median OS and 2-year OS were 12 (95% CI 13.7–NA) months and 60.5% (95% CI 43.7–83.8%), respectively. Pair-wise log rank p value was significant between mucosal and cutaneous (P < 0.001) with better EFS and OS for cutaneous group, mucosal and uveal (P = 0.044) with better EFS and OS for the uveal group, and insignificant between cutaneous and uveal (P = 0.431).

Patients with cutaneous melanomas had superior EFS [HR: 0.78 (95% CI: 0.62–0.98, P = 0.029] (**Figure 2A**) and superior OS (**Figure 2B**) [HR: 0.65 (95% CI: 0.51–0.82, P < 0.001)] compared to those with mucosal melanoma. Patients with uveal melanomas fared better *versus* those with mucosal melanoma, with respect to EFS [HR: 0.68 (95% CI: 0.39–1.3, P = 0.192] and OS [HR: 0.53 (95% CI: 0.29–0.95, P = 0.033)].

#### *BRAF* Mutation

Out of 46 for whom *BRAF* data is available, 24 (52.2%) were cutaneous, 21 (45.7%) were mucosal, and 1 (2.2%) was uveal. Amongst, 95.2% (n = 20) of the mucosal were wild type and 1 was uninterpretable; 79.2% (n = 19) of the cutaneous were wild type, while 20.8% (n = 5) were mutated and the only a single case of uveal melanoma was of wild type.

#### Outcomes With Different Treatment Patterns in Mucosal Cohort (n = 336)

**Figure 3:**
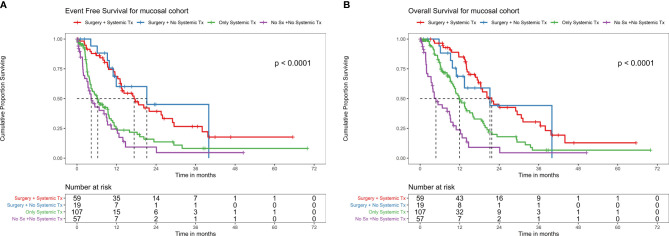
**(A)** EFS with different treatment patterns in the mucosal melanoma cohort. EFS with different treatment patterns (Tx) in the mucosal melanoma cohort. Among patients who underwent surgery and systemic therapy as neoadjuvant chemotherapy (NACT) (n = 59), there were 36 events and the median EFS was 17.3 months (95% CI 9.6–23.4) and 2-year EFS was 39.3% (95% CI 27.3–56.6%). Among patients who underwent surgery without systemic therapy as NACT (n = 19), there were eight events and the median EFS and 2-year EFS were 21.2 months (95% CI 11–NA) and 45.1% (95% CI 22.3–91.5%), respectively. Among patients who did not undergo surgery (due to unresectable locally advanced disease or metastatic disease or unfit for surgery due to patient comorbidities/poor performance status), systemic therapy alone (n = 107) had 69 events, where the median EFS and 2-year EFS were 6.31 months (95% CI 4.3–9.5) and 13.7% (95% CI 7.2–26.1%), respectively. Among patients who did not undergo surgery or receive systemic therapy (due to comorbidities or poor performance status) (n = 57), there were 34 events, the median EFS was 4.3 months (95% CI 3.3–9.3), and 2-year EFS was 9.3% (95% CI 2.8–30.3%). **(B)** OS with different treatment patterns in the mucosal melanoma cohort. OS with different treatment patterns in the mucosal melanoma cohort. Among patients who underwent surgery and systemic therapy (ST) as neoadjuvant chemotherapy (NACT) (n = 59), there were 34 deaths with median OS of 21.7 months (95% CI 18.4–31.2) and 2-year OS of 42.6% (95% CI 30.0–60.5%).Among patients who underwent surgery without ST as NACT (n = 19), there were eight deaths with median OS of 21.2 months (95% CI 11–NA) and 2-year OS of 44.2% (95% CI 21.5–91.1%). Among patients who did not undergo surgery (due to unresectable locally advanced disease or metastatic disease or unfit for surgery due to patient comorbidities/poor ECOG-performance status (PS), ST alone (n = 107)), there were 62 deaths with the median OS and 2-year OS of 11.9 months (95% CI 10.4–15.9) and 18.0% (95% CI 10.4–31.2%) respectively. Among patients who did not undergo surgery or receive ST (due to comorbidities or poor ECOG-PS) (n = 57), the median OS was 4.8 months (95% CI 3.6–9.3) with 34 deaths and 2-year OS of 9% (95% CI 2.8–29.7%).

There were 278 non-metastatic and 58 were metastatic patients. Among those who underwent surgery and systemic therapy as neoadjuvant chemotherapy (NACT) or adjuvant (n = 59), there were 36 events and the median EFS was 17.3 months (95% CI 9.6–23.4) and 2-year EFS was 39.3% (95% CI 27.3–56.6%) (**Figure 3A**). There were 34 deaths with median OS of 21.7 months (95% CI 18.4–31.2) and 2-year OS of 42.6% (95% CI 30.0–60.5%) (**Figure 3B**).

Among patients who underwent surgery without ST (n = 19), there were eight events and the median EFS and 2-year EFS were 21.2 months (95% CI 11–NA) and 45.1% (95% CI 22.3–91.5%), respectively (**Figure 3A**). There were eight deaths with median OS of 21.2 months (95% CI 11–NA) and 2-year OS of 44.2% (95% CI 21.5–91.1%) (**Figure 3B**).

Among patients who did not undergo surgery (due to unresectable locally advanced disease or metastatic disease or unfit for surgery due to patient comorbidities/poor performance status), ST alone (n = 107) had 69 events with the median EFS and 2-year EFS were 6.31 months (95% CI 4.3–9.5) and 13.7% (95% CI 7.2–26.1%), respectively (**Figure 3A**). There were 62 deaths with the median OS and 2-year OS of 11.9 months (95% CI 10.4–15.9) and 18.0% (95% CI 10.4–31.2%), respectively (**Figure 3B**).

Among patients who did not undergo surgery or receive ST (due to comorbidities or poor ECOG-performance status (PS)) (n = 57), there were 34 events. The median EFS was 4.3 (95% CI 3.3–9.3) months, and 2-year EFS was 9.3% (95% CI 2.8–30.3%) (**Figure 3A**). The median OS was 4.8 (95% CI 3.6–9.3) months with 34 deaths and 2-year OS of 9% (95% CI 2.8–29.7%) (**Figure 3B**).

#### Outcomes With Different Treatment Patterns for Cutaneous Cohort (n = 294)

**Figure 4:**
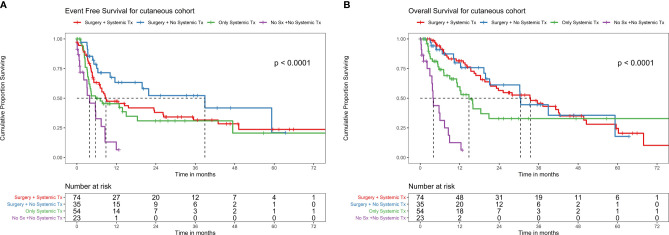
**(A)** EFS with different treatment patterns in the cutaneous melanoma cohort. EFS with different treatment patterns in the cutaneous melanoma cohort. Among patients who underwent surgery and systemic therapy (ST) as neoadjuvant chemotherapy (NACT) (n = 74), there were 46 events, the median EFS was 8.8 months (95% CI 7.1–26.3), and 2-year EFS was 38% (95% CI 27.6–52.3%). Among patients who underwent surgery without ST as NACT (n = 35), there were 15 events, the median EFS was 38.9 months (95% CI 11.6–NA), and 2-year EFS was 52.2% (95% CI 35.5–76.9%). Among patients who did not undergo surgery (due to unresectable locally advanced disease or metastatic disease or unfit for surgery due to patient comorbidities/poor ECOG-performance status (PS), ST alone (n = 54) had 32 events, the median EFS was 5.7 months (95% CI 3.7–NA), and 1-year EFS was 6.5% (95% CI 0.9–43.2%). Among patients who did not undergo surgery or receive systemic therapy (due to comorbidities or poor ECOG-PS) (n = 23), there were 16 events, the median EFS was 3.8 months (95% CI 2.1–8.6), and 2-year EFS was 9.3% (95% CI 2.8–30.3%). **(B)** OS with different treatment patterns in the cutaneous melanoma cohort. Among patients who underwent surgery and systemic therapy (ST) as neoadjuvant chemotherapy (NACT) (n = 74), the median OS was 33.5 months (95% CI 21.3–49.7) with 40 deaths and 2-year OS of 56.5% (95% CI 45.1–70.8%). Among patients who underwent surgery without ST as NACT (n = 35), the median OS was 30.5 months (95% CI 21.1–NA) with 15 deaths and 2-year OS of 61.2% (95% CI 44.4–84.3%). Among patients who did not undergo surgery (due to unresectable locally advanced disease or metastatic disease or unfit for surgery due to patient comorbidities/poor ECOG-performance status(PS), ST alone (n = 54), the median OS was 14.8 months (95% CI 11.1–NA) with 24 deaths, and 2-year OS of 32.8% (95% CI 19.4–55.4%). Among patients who did not undergo surgery or receive ST (due to comorbidities or poor ECOG-PS) (n = 23), the median OS was 4.0 months (95% CI 3.6–8.9) with 16 deaths and 1 year OS of 12.5% (95% CI 3.4–45.5%).

There were 74 metastatic patients and 220 non metastatic patients. Among the patients who underwent surgery and ST as NACT (n = 74), there were 46 events, the median EFS was 8.8 (95% CI 7.1–26.3) months, and 2-year EFS was 38% (95% CI 27.6–52.3%) (**Figure 4A**). The median OS was 33.5 (95% CI 21.3–49.7) months with 40 deaths and 2-year OS of 56.5% (95% CI 45.1–70.8%) (**Figure 4B**).

Among patients who underwent surgery without ST as NACT (n = 35), there were 15 events, the median EFS was 38.9 months (95% CI 11.6–NA), and 2-year EFS was 52.2% (95% CI 35.5–76.9%) (**Figure 4A**). Median OS was 30.5 (95% CI 21.1–NA) months with 15 deaths and 2-year OS of 61.2% (95% CI 44.4–84.3%) (**Figure 4B**).

Among patients who did not undergo surgery (due to unresectable locally advanced disease or metastatic disease or unfit for surgery due to patient comorbidities/poor ECOG-PS), ST alone (n = 54) had 32 events, the median EFS was 5.7 months (95% CI 3.7–NA), and 1-year EFS was 6.5% (95% CI 0.9–43.2%) (**Figure 4A**). Median OS was 14.8 months (95% CI 11.1–NA) with 24 deaths and 2-year OS of 32.8% (95% CI 19.4–55.4%) (**Figure 4B**).

Among patients who did not undergo surgery or receive ST therapy (due to comorbidities or poor ECOG-PS) (n = 23), there were 16 events, the median EFS was 3.8 months (95% CI 2.1–8.6), and 2-year EFS was 9.3% (95% CI 2.8–30.3%) (**Figure 4A**). The median OS was 4.0 months (95% CI 3.6–8.9) with 16 deaths and 1 year OS of 12.5% (95% CI 3.4–45.5%) (**Figure 4B**).

#### Uveal Cohort (n = 19)

With surgery and ST (n = 8) with 6 EFS events, the median EFS was 2.3 months (95% CI 1.6–NA) (**Supplementary Figure S1**). Median OS was 52.3 months (95% CI 5.8–NA) with five deaths (**Supplementary Figure S2**).

With surgery and without ST (n = 6) with 1 EFS events, the median EFS was 13.8 months (95% CI 13.8–NA) (**Supplementary Figure S1**). Median OS was 13.7 months (95% CI 13.7–NA) with one death (**Supplementary Figure S2**).

Without surgery and with ST (n = 3) with 2 EFS events, the median EFS was 12.88 months (95% CI 6.8–NA) (**Supplementary Figure S1**). Median OS was 13.7 months (95% CI 7.8–NA) with two deaths (**Supplementary Figure S2**).

Without surgery and without ST (n = 2) with two EFS events, the median EFS was 2.3 months (95% CI 0.23–NA) (**Supplementary Figure S1**). Median OS was 2.6 months (95% CI 0.7–NA) with two deaths (**Supplementary Figure S2**).

### Metastatic Cohort

The commonest metastatic sites were non-regional nodes (n = 178, 61.1%), lung (n = 124, 42.8%), liver (n = 123, 42.4%), bones (n = 55, 19%), peritoneum (n = 27, 9.3%), and soft tissues (n = 39, 13.5%).

The overall metastatic cohort (OMC) (n = 433) (65.7%) comprised baseline metastatic cohort (n = 291), nonmetastatic who develop metastasis during presentation to us (n = 81), and *de novo* nonmetastatic at presentation who subsequently failed distally (n = 61). The median follow-up in OMC was 24 (0–85) months. Two hundred and nineteen patients (50.6%) received ST, while 45 (10.4%) received ICI. One hundred and ten patients (25.4%) were declared BSC, and 59 (13.6%) did not follow. The most common ST regimens were taxanes with or without platinum and/or SC-IFN in 159 (36.7%) followed by ICI in 45 (10.4%), OMCT based in 31 (7.2%), and temozolamide based in 26 (6%) patients. The objective response rate (ORR) to ST was 54 (28.12%), with 3 CRs; additionally, 45 (23.43%) had stable disease adding to the disease control rate of 51.55%; 93 (48.4%) progressed and responses were not available in 72 (**Figure 1**).

### Best Supportive Care *Versus* Any Systemic Therapy in Overall Metastatic Cohort

**Figure 5:**
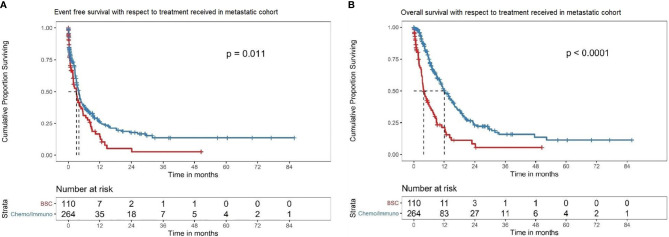
**(A)** EFS in the overall metastatic cohort based on treatment offered. Kaplan–Meier curve showing the EFS of the patients in the overall metastatic cohort subdivided into best supportive care alone (BSC) (red) *versus* any systemic therapy (chemotherapy/immunotherapy/interferons) (blue). In this cohort, the patients with BSC alone had a median EFS of 3.1 (95% CI 1.9–4.8) months, while those treated with any systemic therapy had a median EFS of 3.98 (95% CI 3.2–4.7) months (HR: 0.69, 95%CI: 0.52–0.92; P = 0.011). **(B)** OS in the overall metastatic cohort based on treatment offered. Kaplan–Meier curve showing the OS of the patients in the overall metastatic cohort subdivided into best supportive care alone (BSC) (red) versus any systemic therapy (chemotherapy/immunotherapy/interferons) (blue). In this cohort, the median OS with BSC alone was 3.9 (95% CI 3.3–6.4) months *versus* 12.0 (95% CI 10.5–15.1) months in any systemic therapy group (HR: 0.38, 95% CI: 0.28–0.50; P < 0.001).

The median EFS for BSC was 3.1 (95% CI 1.9–4.8) months *versus* 3.98 (95% CI 3.2–4.7) months with any ST (HR: 0.69, 95% CI: 0.52–0.92; P = 0.011) (**Figure 5A**). The median OS was 3.9 months (95% CI 3.3–6.4) for BSC alone *versus* 12.0 months (95% CI 10.5–15.1) in any ST (HR: 0.38, 95% CI: 0.28–0.50; P < 0.001) (**Figure 5B**)

### Non-Metastatic Cohort (n = 368)

The median follow-up was 26 (0–83) months. Of these, 172 (46.7%) had radical surgery before presentation to our center, of which 81 (47.1%) developed metastasis and remaining 91 (52.9%) continued as non-metastatic at baseline evaluation at our institute. *De novo* non-metastatic cases were 196 (53.3%), of which 142 (76.8%) underwent resection at our institute (anorectal n = 66, extremity n = 31, head and neck n = 19, and other sites n = 26), while 11 (2.9%) patients were unresectable. Among these 66 anorectal cases, 55 (83.3%) underwent abdomino-perineal resection (APR), while remaining underwent trans-anal excision. Pelvic node dissection was performed in 18 (32.72%) patients. Circumferential resection margins were negative in 47 (85.45%) patients.

Adjuvant ICI was offered to 25 (6.8%), adjuvant CT to 8 (2.2%), and adjuvant RT to 78 (11.8%) patients. Median EFS of baseline non-metastatic patients at diagnosis was 29.5 (95% CI: 22–40) months, while median OS was 33.3 months (95% CI: 29.5–41.2); 2-year EFS and OS were 54.4% (95% CI: 48.6–60.9%) and 61.1% (95% CI: 55.2–67.6%), respectively (**Supplementary Figure S3**).

### Safety Results

Commonest grade 3–4 toxicity was anemia with chemotherapy (9.5%) followed by thrombocytopenia (3.6%) and with ICI anemia (8.8%). Toxicity profile is highlighted in **Supplementary Table S1**.

### Prognostic Markers

The factors that were found to be significant for prognosis in the univariate analysis for EFS and OS are shown in **Table 3**. In the overall cohort, for multivariate analysis, surgery performed [HR: 0.34 (95% CI 0.270.43), P = 0.001] and extremity primary [HR: 0.74 (95% CI 0.57–0.97); P = 0.029] were significant factors for superior EFS. For OS, ST offered [HR: 0.46 (95% CI 0.35–0.61); P = 0.001] and surgery performed [HR: 0.40 (95% CI 0.30–0.53); P < 0.001] were significant factors with superior OS (**Table 3**).

In baseline non-metastatic cohort in multivariate analysis for OS, surgical resection [HR 0.38 (95% CI 0.20–0.73) P = 0.001] and ST offered [HR: 0.55 (95% CI 0.35–0.88) P = 0.013] were significant factors for superior outcomes.

## Discussion

In India, cancer registries report that the age-specific incidence rate for cutaneous malignant melanoma is less than 0.5 per 100,000 (16). The highest age-standardized incidence rates (ASIR) per 100,000 general population were reported in Australia (54.1) and United States (21.0), while the lowest ones include Asia Pacific (0.7) and South Asia (1.1) (17).

In our study, 44.2% of patients were metastatic at presentation. Contrastingly, in a West-Asian study, around 12% patients were metastatic at the baseline, and according to the western literature, 4% patients presented with metastasis (18, 19). The higher number of metastatic patients at presentation in our settings is attributed to the paucity of a proper melanoma registry and lack of awareness in patients and community practitioners about melanoma diagnosis. Moreover, logistic constraints in LMIC leading to delayed referral and upstaging also contributes for the same (15, 20, 21). In the present study, the most common metastatic site was non- regional nodes (61.1%), in contrast to a western study, wherein lung constituted the predominant metastatic site, in 85% cases (22). This is partially explained by the differential distribution of primary sites and types. However, there might be pharmaco-genomic variations as well, which are largely unexplored.

The median age was 54 years, with 58.9% of males. This correlates with the European, Australian, and other Asian countries where men are more susceptible to melanoma than females, and the median age is in the 5^th^ decade (2, 23). It is important to note that taking age into consideration, adolescents and young adult women are more susceptible to melanoma than men are, but after the age of 40 years, the pattern reverses with a relatively higher prevalence in men (2).

In the current study, the most common site was extremity (36.6%), followed by anorectal region (31.4%). Overall, 55.2% of patients had mucosal melanoma, while 44.8% had cutaneous primary. This contrasts with the data from tropical countries and other parts of the world (22, 24). In a Eurocare-5 study report, 15% of the patients had cutaneous melanoma of the head and neck region (24). In other Indian studies also, mucosal melanomas were more common. However, there is a variable pattern that is partly explained by referral pattern and draining area (24). Notably, ophthalmic melanoma was reported as the most common in a study from a leading ophthalmology referral center. In other study, albeit with small numbers, anorectal and extremity were the predominant sites (43% for both) (25). Among various histopathological subtypes, nodular melanoma was the most common in our study. However, in other Asian and western studies, the superficial spreading type was more common. Considering ours as a cancer referral centre, we receive tumours and lesions of larger sizes (18, 24).

Among the limited patients who were tested, the incidence of *BRAF* mutation was 10.8%, which is comparable to other Asian data (15–25%). However, this is lower than the western literature (45%) (26, 27). In another Indian study, the incidence of *BRAF* mutations was around 30% (28). *BRAF* is less common in mucosal melanomas than cutaneous melanomas, and this analysis cohort had a higher proportion of mucosal melanoma and this could partly explain the low prevalence of *BRAF* mutations. Notably none of the tested mucosal or uveal melanoma were *BRAF* mutation positive. This small sample artifact partly explains this, and the entire cohort needs testing to know the actual incidence of *BRAF* mutations in India.

In spite of the lack of standard of care options, such as ICI and/or targeted therapy, there was a statistically significant improvement in EFS and OS in the metastatic cohort with any ST in comparison to the BSC alone. In real world practice, other therapies such as LD-SCIFN and OMCT were used in some patients, which has immunomodulatory properties. One can argue that there might be higher poor ECOG PS patients in BSC rather than in ST arm; however, a significant number of patients with advanced disease in real world scenario of LMIC present with high tumour burden, brain metastasis, nutritional deficiencies, extremes of ages, and poor ECOG PS. Although we do not have exact numbers of patients who presented with poor PS in each group owing to inadequate documentation of this factor in all patients, this could be a potential confounder owing to inherent limitation of retrospective analysis. Among various treatment options other than immunotherapy and targeted therapy, the disease control rates and median PFS were better with paclitaxel and carboplatin with or without low dose subcutaneous interferon compared to temozolamide based regimens as shown in **Figure 1**. The survival statistics seem comparable with these regimens, in comparison to other regimens used in literature, and are worth exploring systematically (29) (**Table 4**).

**Table 4:** Studies of systemic chemotherapy in metastatic melanoma.

Study	Sites	Drug used	No. of patients		ORR (%)	Median OS
Pflugfelder et al. (30)	All	Paclitaxel + Carboplatin	61		4.9	31 weeks
Anderson et al. (31)	All	Cisplatin, Vinblastine and Dacarbazine *Vs*.Dacarbazine	46 *vs* 45		24 *vs* 11	6 *vs* 5 months
Falkson et al. (32)	All	Dacarbazine + interferon alfa *Vs*. dacarbazine	31 *vs* 30		52 *vs* 50	7.2 *vs* 4.8 months
Bajpai J et al. (current study)	All	Best Supportive care *vs* any systemic therapy (Pl+ C, Interferon, P+C+interferon; OMCT, TMZ, DTIC, others)	433 of 649 total			BSC - 4.27 (95% CI: 3.06-7.4) months ST- 12.5 months (95% CI: 11-16.3) months (HR: 0.32, 95% CI: 0.22-0.45; P<0.001)
**Studies according to site**						
Ranjith et al. (33)	Anorectal	Dacarbazine, Temozolamide + Thalidomide	22		–	9 months for metastatic 15 months for non metastatic
Chen et al. (34)	Anorectal	Best supportive care *Vs*. Chemotherapy (Dacarbazine, Oral metronomic therapy)	12 *Vs*. 25		0 *Vs*. 24	14 weeks *Vs*. 33 weeks

P+C, Paclitaxel and carboplatin; DTIC, dacarbazine; Interferon-subcutaneous, low dose interferon; OMCT, oral metronomic chemotherapy; TMZ, Temozolamide; BSC, best supportive care.

A large majority of patients from this part of the world is self-paying. A monthly therapy with either pembrolizumab, 200 mg 3 weekly schedule or for nivolumab 3 mg/kg, two weekly schedules for an average-weight person costs nearly 5000 USD, which is beyond reach to a vast majority of patients in LMIC (35, 36). India’s existing data suggest that only 1.6% of the eligible patients could afford ICI, which reflects the gross discrepancies between higher-income countries *versus* LMIC. Studies from even developed countries, such as Australia and developing countries, have concluded that rapidly rising treatment costs of melanoma warrant an urgent need for a comprehensive melanoma control strategy (30, 31).

The survival in patients harbouring cutaneous melanoma was superior compared to mucosal melanoma, which is similar to published literature (32–34). Site-wise, cases with extremity tumors fared much better than others, and anorectal melanomas had the worst outcomes, as similarly reported by others (32–34, 37). Anorectal melanoma is known to have a very aggressive behaviour (38, 39). **Table 4** highlights various studies of ST for the management of MM.

Despite being one of the few studies from our subcontinent, this study has several limitations, including nonrandomized character with inherent biases; our institute being the tertiary care referral centre, there is a chance of referral bias, and this may not represent the true population, including the primary site and subtypes. However, this is the most extensive data from India, where melanoma is a rare diagnosis and can provide meaningful inferences.

Only 10% of patients received the standard of care options, including ICI and targeted therapies due to financial constraints. However, this is the real-world data and mirrors the actual practice.

Malignant melanoma is a rare disease in this part of the world and has a dismal prognosis without ICI or targeted therapy. However, meaningful responses can be achieved with other systemic therapies, if these standard options are not feasible (40–42). There is an urgent need to have a national registry, national and international collaborations for clinical trials, and patients support programs to increase access to the standard therapy. However, till then, other STs including those with immunomodulatory potential, such as chemo with LD-IFN and OMCTs, are worth exploring as an option. The efficacy of these systemic therapy options needs further validation in prospective studies and caution should be exercised in correct interpretation of the results as these are certainly not the substitute for standard of care therapies.

Large real-world data reflects the treatment patterns adopted in a LMIC for melanomas and the hard reality of poor access to expensive standard of care therapies. In such real-world situations, when standard options are beyond reach, other systemic therapies may provide meaningful clinical benefit and are worth considering.

## Data Availability Statement

The raw data supporting the conclusions of this article will be made available by the authors, without undue reservation.

## Ethics Statement

The study was conducted after approval from the Institutional Ethics Committee (IEC). Waiver of consent was obtained for retrospective study. All data were anonymized before the start of analysis.

## Author Contributions

JB, AS, and SB did conceptualization. JB, GA, AS, AA, SD, AmC,AK, PE, PM, PB, SS, ST, ArC BR, NK, AJ, AKJ, MB, VO, AR, JR, AD, AG, RK, NM, SR, VP, VN, AJ, SL, VR, KP, SG, and SB contributed to the methodology. Software work management done by JB, GA, and AA. Validation done by JB, GA, AA, SG, and SB. Formal analysis done by JB, GA, AA, and SG. Resources provided by JB, SG, and SB. Data curation done by JB, GA, AA, SD, PE, PM, AmC, and SB. Original draft written by JB, GA, AA, and AK. Review and editing done by JB, GA, ArC, BR, SG, SB, and KP. Visualisation done by JB, GA, AA, SB, and AS. The project was supervised by JB, SB, SG, BR, AS, KP, SL, and VR. Project aquisition done by JB, AS, SG, KP, and SB. All authors contributed to the article and approved the submitted version.

## Conflict of Interest

The authors declare that the research was conducted in the absence of any commercial or financial relationships that could be construed as a potential conflict of interest.

## Publisher’s Note

All claims expressed in this article are solely those of the authors and do not necessarily represent those of their affiliated organizations, or those of the publisher, the editors and the reviewers. Any product that may be evaluated in this article, or claim that may be made by its manufacturer, is not guaranteed or endorsed by the publisher.

## Supplementary Material

Supplementary Figure 1 The EFS with different treatment patterns (Tx) in uveal melanoma. With surgery and with systemic therapy (ST) as neoadjuvant therapy (NACT) (n = 8) with 6 EFS events, the median EFS was 2.3 months (95% CI 1.6–NA). With surgery and without ST NACT (n = 6) with 1 EFS events, the median EFS was 13.8 months (95% CI 13.8–NA). Without surgery and with systemic therapy as NACT (n = 3) with 2 EFS events, the median EFS was 12.88 months (95% CI 6.8–NA). Without surgery and without ST as NACT (n = 2) with 2 EFS events, the median EFS was 2.3 months (95% CI 0.23–NA).

Supplementary Figure 2 OS with different treatment patterns (Tx) in uveal melanoma. With surgery and with systemic therapy (ST) as neoadjuvant therapy (NACT) (n = 8), the median OS was 52.3 months (95% CI 5.8–NA) with 5 deaths. With surgery and without systemic therapy as NACT (n = 6), the median OS was 13.7 months (95% CI 13.7–NA) with 1 death. Without surgery and with ST as NACT (n = 3), the median OS was 13.7 months (95% CI 7.8–NA) with 2 deaths. Without surgery and without systemic therapy as NACT (n = 2), the median OS was 2.6 months (95% CI 0.7–NA) with 2 deaths.

Supplementary Figure 3 Showing the outcome parameters of EFS (red) and OS (blue) in the baseline non metastatic cohort. Median EFS of non-metastatic patients was 29.5 (95% CI: 22–40) months, while median OS was 33.3 months (95% CI: 29.5–41.2); 2-year EFS and OS were 54.4% (95% CI: 48.6–60.9%) and 61.1% (95% CI:55.2–67.6%), respectively.
