# Supplementary figures and images for: Demographics, Pattern of Care, and Outcome Analysis of Malignant Melanomas - Experience From a Tertiary Cancer Centre in India
Source: Front Oncol. 2021 Sep 8;11:710585. doi: 10.3389/fonc.2021.710585 (PMC8456006; doi:10.3389/fonc.2021.710585)

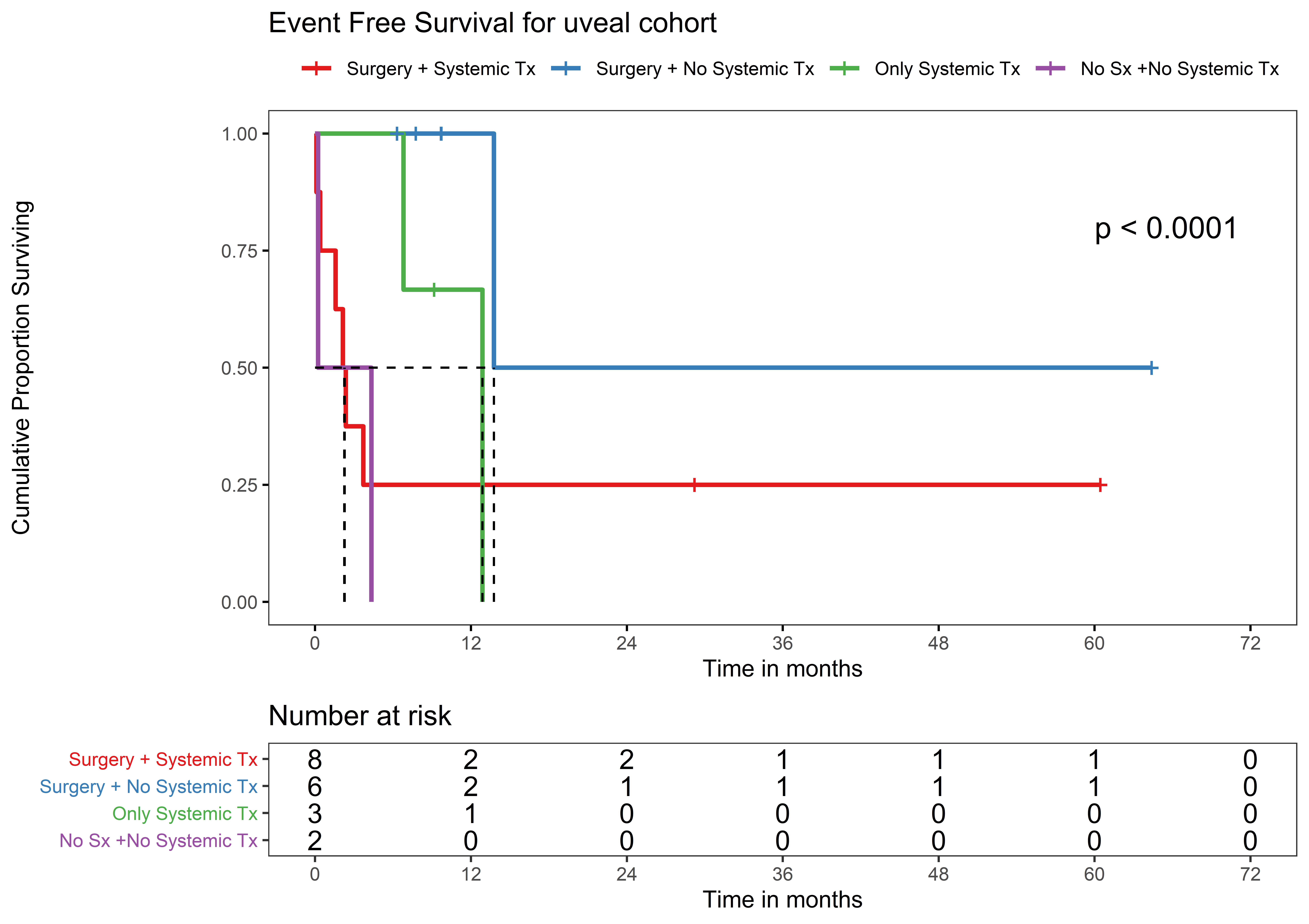

Supplement: Supplementary Figure 1 — The EFS with different treatment patterns (Tx) in uveal melanoma. With surgery and with systemic therapy (ST) as neoadjuvant therapy (NACT) (n = 8) with 6 EFS events, the median EFS was 2.3 months (95% CI 1.6–NA). With surgery and without ST NACT (n = 6) with 1 EFS events, the median EFS was 13.8 months (95% CI 13.8–NA). Without surgery and with systemic therapy as NACT (n = 3) with 2 EFS events, the median EFS was 12.88 months (95% CI 6.8–NA). Without surgery and without ST as NACT (n = 2) with 2 EFS events, the median EFS was 2.3 months (95% CI 0.23–NA). [file Image_1.jpeg]

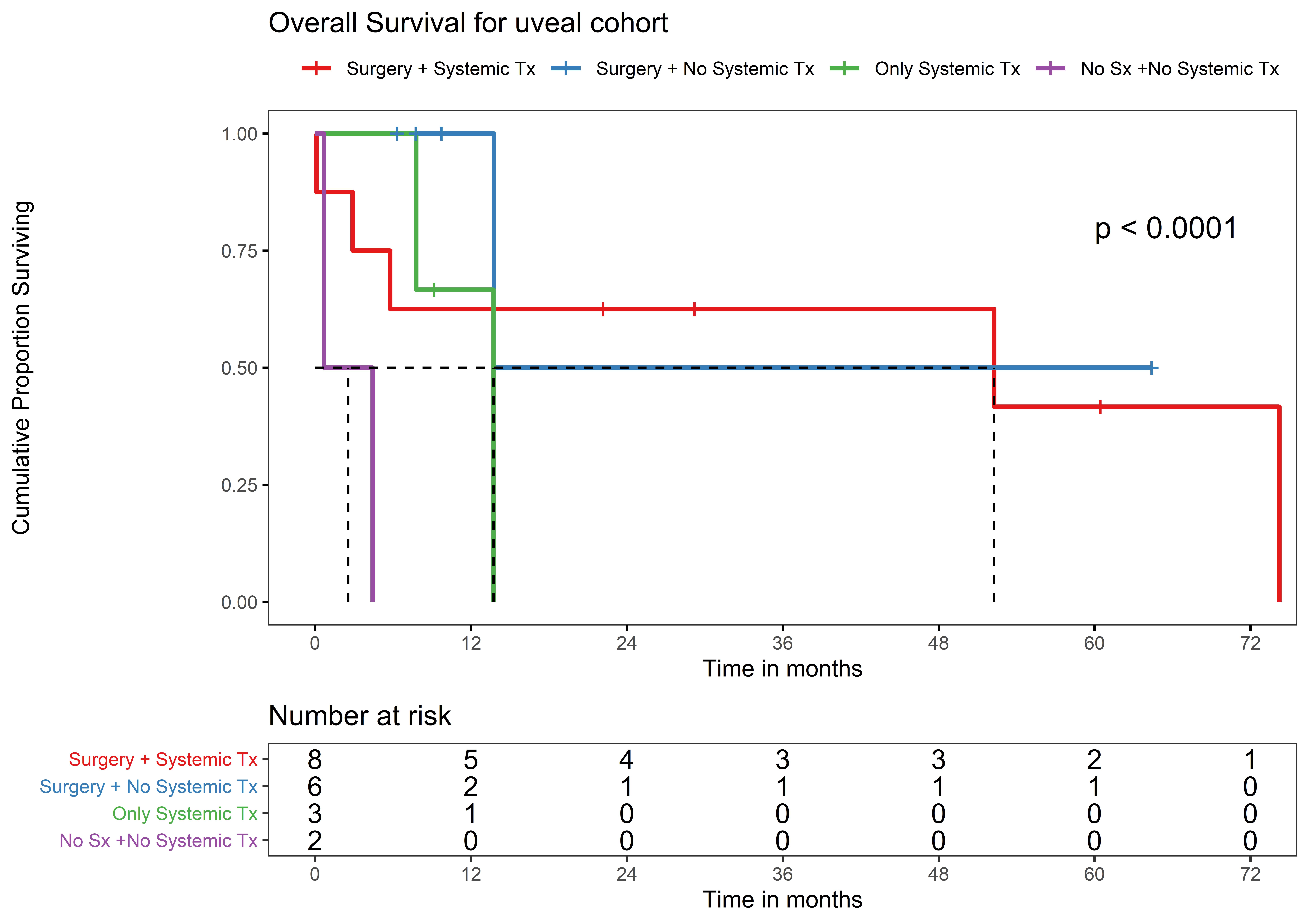

Supplement: Supplementary Figure 2 — OS with different treatment patterns (Tx) in uveal melanoma. With surgery and with systemic therapy (ST) as neoadjuvant therapy (NACT) (n = 8), the median OS was 52.3 months (95% CI 5.8–NA) with 5 deaths. With surgery and without systemic therapy as NACT (n = 6), the median OS was 13.7 months (95% CI 13.7–NA) with 1 death. Without surgery and with ST as NACT (n = 3), the median OS was 13.7 months (95% CI 7.8–NA) with 2 deaths. Without surgery and without systemic therapy as NACT (n = 2), the median OS was 2.6 months (95% CI 0.7–NA) with 2 deaths. [file Image_2.jpeg]

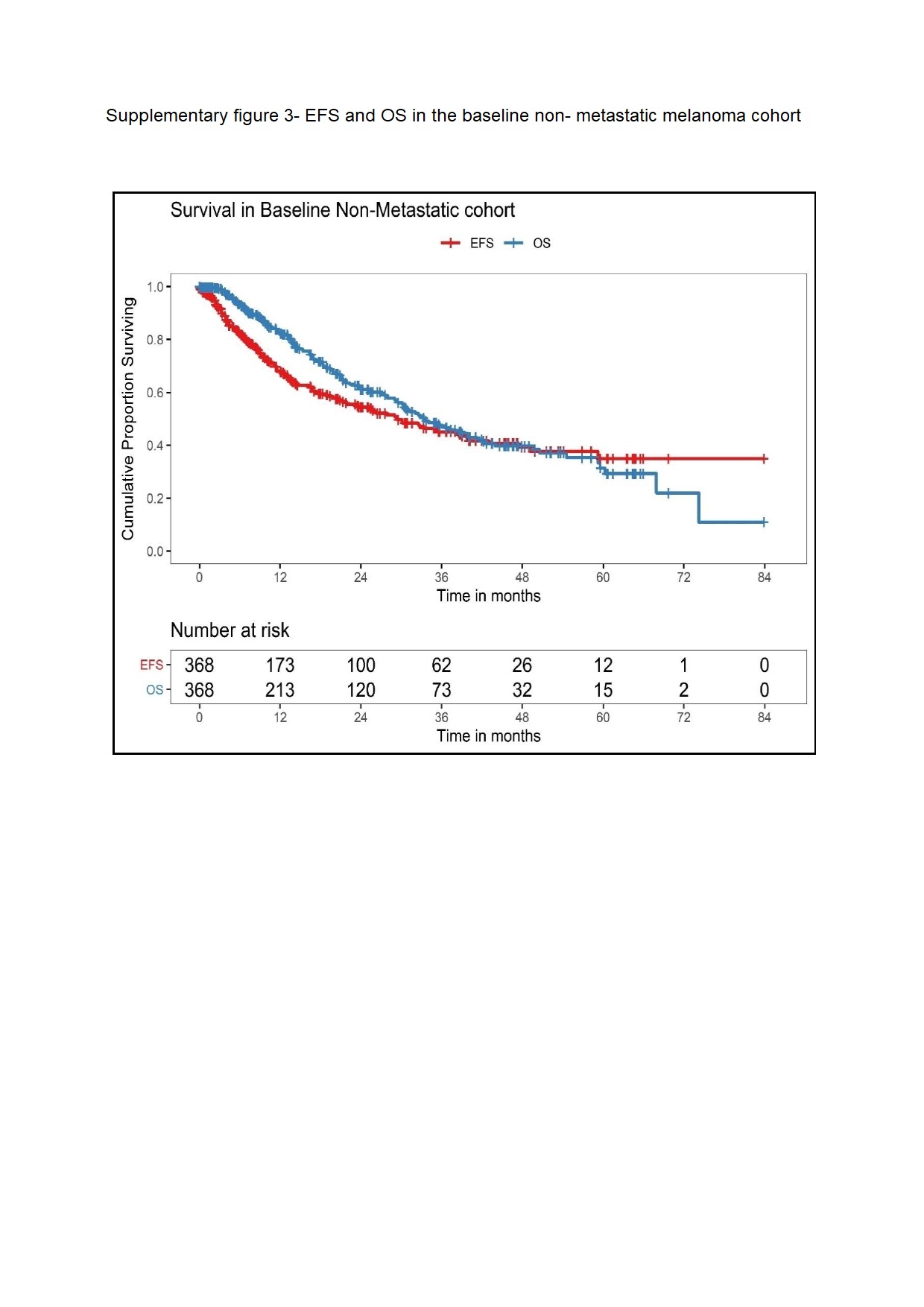

Supplement: Supplementary Figure 3 — Showing the outcome parameters of EFS (red) and OS (blue) in the baseline non metastatic cohort. Median EFS of non-metastatic patients was 29.5 (95% CI: 22–40) months, while median OS was 33.3 months (95% CI: 29.5–41.2); 2-year EFS and OS were 54.4% (95% CI: 48.6–60.9%) and 61.1% (95% CI:55.2–67.6%), respectively. [file Image_3.jpeg]
