# Supplementary material for: Demographics, Pattern of Care, and Outcome Analysis of Malignant Melanomas - Experience From a Tertiary Cancer Centre in India
Source: Front Oncol. 2021 Sep 8;11:710585. doi: 10.3389/fonc.2021.710585 (PMC8456006; doi:10.3389/fonc.2021.710585)
Supplement: Supplementary file 4 [file Table_1.docx]

#### **Supplementary table 1- Univariate and multivariate analysis for EFS and OS (Only significant factors)**

|  | | **Univariate and Multivariate analysis for EFS (Only significant factors)** | | | | | | | |  | |  |
| --- | --- | --- | --- | --- | --- | --- | --- | --- | --- | --- | --- | --- |
|  |  | **Overall cohort (N=659)** | | **Baseline Non-Metastatic (N=368)** | | | **Baseline Metastatic (N=291)** | | | **Overall Metastatic (baseline + relapsed) (N= 433)** | | |
|  | **N** | **Univariate** | **Multivariate** | **N** | **Univariate** | **Multivariate** | **N** | **Univariate** | **Multivariate** | **N** | **Univariate** | **Multivariate** |
| **Prognostic factor** |  | **HR (95%CI)**  **; P value** |  |  | **HR (95%CI) ; P value** |  |  | **HR (95%CI)**  **; P value** |  |  | **HR (95%CI)**  **; P value** |  |
| **Site of**  **Primary** |  |  |  |  |  |  |  |  |  |  |  |  |
| Anorectal | 207 | (Ref.) | (Ref.) | 91 | (Ref.) |  | 116 |  |  | 160 |  |  |
| Extremities | 241 | 0.68 (0.52-  0.88) ;0.003 | 0.83 (0.63-  1.08) ;0.165 | 156 | 0.82 (0.56-  1.22) ;0.334 |  | 85 | 0.74 (0.52-  1.07) ;0.112 |  | 147 | 0.82 (0.62-  1.10) ;0.181 |  |
| Others | 211 | 0.71 (0.55-  0.93) ;0.012 | 0.74 (0.57-  0.97) ;0.029 | 121 | 0.81 (0.54-  1.22) ;0.312 |  | 90 | 0.74 (0.52-  1.06) ;0.102 |  | 126 | 0.89 (0.67-  1.20) ;0.452 |  |
| **Surgery** |  |  |  |  |  |  |  |  |  |  |  |  |

| No | 308 | (Ref.) | (Ref.) | 64 | (Ref.) |  |  |  |  |  |  |  |
| --- | --- | --- | --- | --- | --- | --- | --- | --- | --- | --- | --- | --- |
| Yes | 351 | 0.34 (0.27-  0.43) ;0.000 | 0.34 (0.27-  0.43) ;0.000 | 304 | 0.42 (0.28-  0.62) ;0.000 |  |  |  |  |  |  |  |
|  |  |  |  |  |  |  |  |  |  |  |  |  |
| **Mucosal Vs.**  **Cutaneous** |  |  |  |  |  |  |  |  |  |  |  |  |
| Mucosal | 364 | (Ref.) | (Ref.) | 185 | (Ref.) |  | 179 |  |  | 248 | (Ref.) | (Ref.) |
| Cutaneous | 295 | 0.81 (0.65-  1.01) ;0.057 |  | 183 | 0.84 (0.61-  1.16) ;0.284 |  | 112 | 0.91 (0.67-  1.24) ;0.555 |  | 185 | 0.89 (0.69-  1.13) ;0.336 |  |
| **Therapy offered** |  |  |  |  |  |  |  |  |  |  |  |  |
| BSC | 123 |  |  | 50 |  |  | 73 |  |  | 110 |  |  |
| Any ST | 305 | 0.77 (0.58-  1.00) ;0.052 |  | 123 | 0.99 (0.63-  1.55) ;0.959 |  | 182 | 0.58 (0.41-  0.81) ;0.001 |  | 264 | 0.89 (0.67-  1.18) ;0.404 |  |
| **Univariate and Multivariate analysis for OS (Only significant factors)** | | | | | | | | | | | | |
| **Site of**  **primary** |  |  |  |  |  |  |  |  |  |  |  |  |
| Anorectal (Ref.) | 207 |  |  | 91 |  |  | 116 |  |  | 160 |  |  |
| Extremities | 241 | 0.55 (0.42-  0.72) ;0.000 | 0.65 (0.36-  1.20) ;0.168 | 156 | 0.71 (0.47-  1.08) ;0.109 |  | 85 | 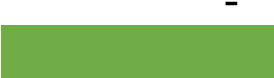0.62 (0.42  0.92) ;0.016 | 0.71 (0.47-  1.08) ;0.110 | 147 | 0.60 (0.45-  0.82) ;0.001 | 0.49 (0.26-  0.91) ;0.024 |
| Others | 211 | 0.63 (0.48-  0.84) ;0.001 | 0.73 (0.51-  1.05) ;0.090 | 121 | 0.80 (0.52-  1.24) ;0.320 |  | 90 | 0.62 (0.43-  0.90) ;0.011 | 0.66 (0.45-  0.99) ;0.045 | 126 | 0.75 (0.55-  1.02) ;0.067 | 0.73 (0.50-  1.07) ;0.104 |
| **Therapy offered** |  |  |  |  |  |  |  |  |  |  |  |  |
| BSC | 123 | (Ref.) | (Ref.) | 50 |  |  | 73 |  |  | 110 | (Ref.) | (Ref.) |
| Any ST | 305 | 0.48 (0.37-  0.64) ;0.000 | 0.46 (0.35-  0.61) ;0.000 | 123 | 0.60 (0.38-  0.95) ;0.031 | 0.55 (0.35-  0.88) ;0.013 | 182 | 0.32 (0.22-  0.46) ;0.000 | 0.35 (0.24-  0.50) ;0.000 | 264 | 0.41 (0.32-  0.53) ;0.000 | 0.54 (0.40-  0.72) ;0.000 |
| **Surgery** |  |  |  |  |  |  |  |  |  |  |  |  |
| No | 308 | (Ref.) | (Ref.) | 64 | (Ref.) |  |  |  |  |  |  |  |
| Yes | 351 | 0.28 (0.22-  0.35) ;0.000 | 0.40 (0.30-  0.53) ;0.000 | 304 | 0.34 (0.22-  0.51) ;0.000 | 0.38 (0.20-  0.73) ;0.004 |  |  |  |  |  |  |
| **Mucosal Vs.**  **Cutaneous** |  |  |  |  |  |  |  |  |  |  |  |  |
| Mucosal | 364 |  | (Ref.) | 185 |  |  | 179 |  |  | 248 |  | (Ref.) |
| Cutaneous | 295 | 0.70 (0.55-  0.88) ;0.002 | 1.05 (0.63-  1.75) ;0.849 | 183 | 0.74 (0.53-  1.04) ;0.082 |  | 112 | 0.82 (0.59-  1.13) ;0.220 |  | 185 | 0.70 (0.54-  0.90) ;0.006 | 1.12 (0.66-  1.91) ;0.669 |

Abbreviations: Ref: reference category, BSC-best supportive care, ST-Systemic therapy
