# Supplementary material for: Demographics, Pattern of Care, and Outcome Analysis of Malignant Melanomas - Experience From a Tertiary Cancer Centre in India
Source: Front Oncol. 2021 Sep 8;11:710585. doi: 10.3389/fonc.2021.710585 (PMC8456006; doi:10.3389/fonc.2021.710585)
Supplement: Supplementary file 5 [file Table_2.docx]

**Supplementary table 2 -Incidence of grade 3 or 4 toxicities associated with chemotherapy and immunotherapy**

| Toxicites (grade 3 or 4) | Chemotherapy cohort n=219  Number (percentage) | Immunotherapy cohort n=45  Number(percentage) |
| --- | --- | --- |
| Anemia | 21 (9.5 %) | 4 (8.8 %) |
| Thrombocytopenia | 8(3.6%) | 0 |
| Neutropenia | 4 (1.8%) | 0 |
| Fatigue | 5 (2.2 %) | 2(4.4%) |
| Hyponatremia | 5(2.2 %) | 3(6.6%) |
| Dysphagia | 5(2.2 %) | 0 |
| Mucositis | 3(1.3 %) | 0 |
| Transaminitis | 2(0.9 %) | 0 |
| Diarrhoea | 2(0.9 %) | 0 |
| Vomiting | 2(0.9 %) | 0 |
| Skin rash | 0 | 2 (4.4 %) |
| Hypothyroidism | 0 | 1(2.2 %) |
| Hyperthyroidism | 0 | 1(2.2 %) |
